# 1-(2-Chloro­acet­yl)-3-methyl-2,6-diphenyl­piperidin-4-one

**DOI:** 10.1107/S1600536809045358

**Published:** 2009-11-04

**Authors:** F. Nawaz Khan, P. Nithya, V. Krishna Kumar, Venkatesha R. Hathwar, Seik Weng Ng

**Affiliations:** a Chemistry Division, School of Science and Humanities, VIT University, Vellore 632 014, Tamil Nadu, India; b Solid State and Structural Chemistry Unit, Indian Institute of Science, Bangalore 560 012, Karnataka, India; c Department of Chemistry, University of Malaya, 50603 Kuala Lumpur, Malaysia

## Abstract

The asymmetric unit of the title compound, C_20_H_20_ClNO_2_, contains two crystallographically independent mol­ecules of similar geometry. The piperidine ring adopts a distorted boat conformation in both mol­ecules, in which the N atom assumes an almost planar configuration.

## Related literature

For the crystal structure of 3,5-dimethyl-bis­(2-methoxy­phen­yl)piperidin-4-one, see: Parthiban *et al.* (2008 ▶).
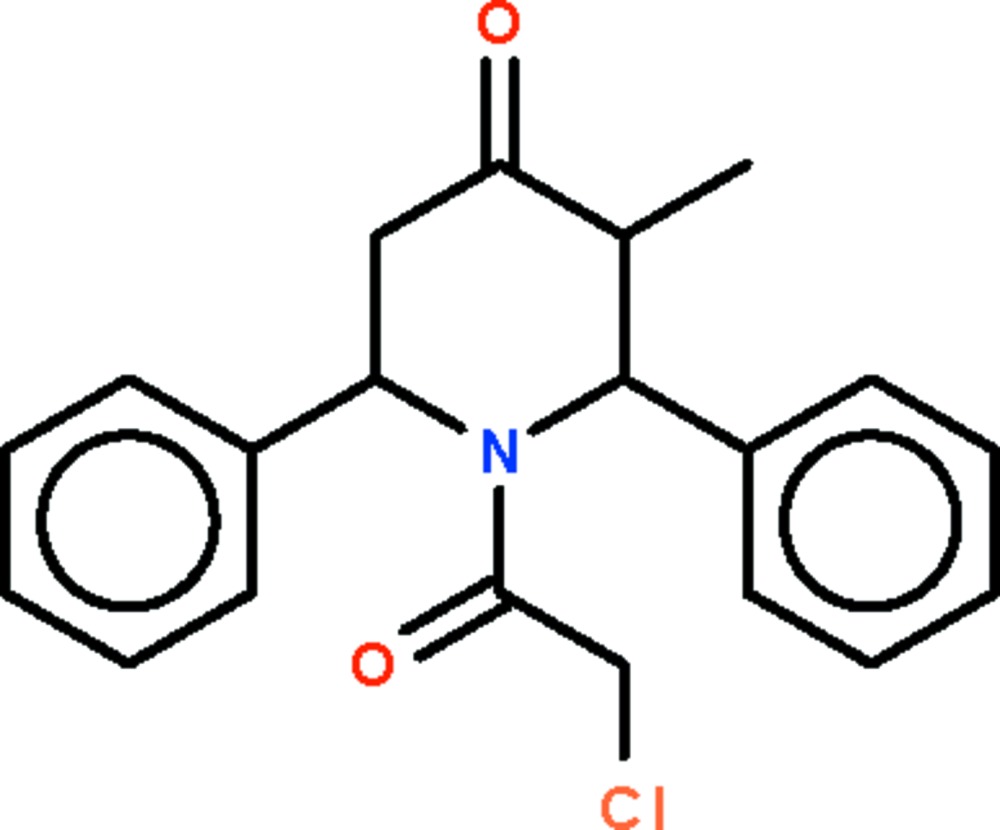


## Experimental

### 

#### Crystal data

C_20_H_20_ClNO_2_
*M* *_r_* = 341.82
Monoclinic, 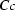

*a* = 31.026 (6) Å
*b* = 12.417 (2) Å
*c* = 9.3209 (17) Å
β = 101.423 (4)°
*V* = 3519.8 (11) Å^3^
*Z* = 8
Mo *K*α radiation
μ = 0.23 mm^−1^
*T* = 290 K
0.25 × 0.23 × 0.20 mm

#### Data collection

Bruker SMART CCD area-detector diffractometer
Absorption correction: multi-scan (*SADABS*; Sheldrick, 1996 ▶) *T*_min_ = 0.945, *T*_max_ = 0.956
14797 measured reflections
7013 independent reflections
4933 reflections with *I* > 2σ(*I*)
*R*_int_ = 0.036

#### Refinement

*R*[*F* ^2^ > 2σ(*F* ^2^)] = 0.051
*wR*(*F* ^2^) = 0.094
*S* = 1.05
7013 reflections
435 parameters
2 restraints
H-atom parameters constrained
Δρ_max_ = 0.31 e Å^−3^
Δρ_min_ = −0.14 e Å^−3^
Absolute structure: Flack (1983 ▶), 3002 Friedel pairs
Flack parameter: 0.04 (5)

### 

Data collection: *SMART* (Bruker, 2004 ▶); cell refinement: *SAINT* (Bruker, 2004 ▶); data reduction: *SAINT*; program(s) used to solve structure: *SHELXS97* (Sheldrick, 2008 ▶); program(s) used to refine structure: *SHELXL97* (Sheldrick, 2008 ▶); molecular graphics: *X-SEED* (Barbour, 2001 ▶); software used to prepare material for publication: *publCIF* (Westrip, 2009 ▶).

## Supplementary Material

Crystal structure: contains datablocks global, I. DOI: 10.1107/S1600536809045358/ci2956sup1.cif

Structure factors: contains datablocks I. DOI: 10.1107/S1600536809045358/ci2956Isup2.hkl

Additional supplementary materials:  crystallographic information; 3D view; checkCIF report

## Figures and Tables

**Fig. 1.**
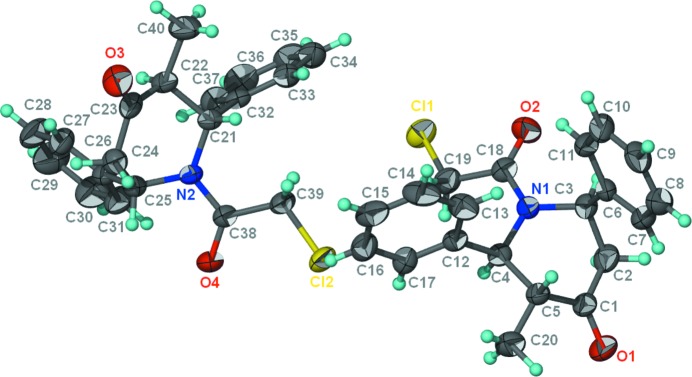
Displacement ellipsoid plot (Barbour, 2001) of C20H20ClNO2 at the 50% probability level. H atoms are drawn as spheres of arbitrary radius.

## supplementary crystallographic information

### Experimental

To a solution of 3-methyl-2,6-diphenylpiperidin-4-one (0.005 mol) and triethylamine (0.005 mol) dissolved in benzene (50 ml), chloroacetyl chloride (0.005 mol) dissolved in benzene (10 ml) was added. The mixture was stirred for an hour. The mixture was then poured into water and the organic product extracted with ether. The ether phase was washed with 3% sodium bicarbonate solution and then dried over anhydrous sodium sulfate. The compound was purified by recrystallization from ethanol.

### Refinement

C-bound H-atoms were placed in calculated positions (C-H = 0.93–0.98 Å) and were included in the refinement in the riding model approximation, with *U_iso_*(H) set to 1.2–1.5*U_eq_*(C).

### Crystal data

**Table:**

C_20_H_20_ClNO_2_	*F*(000) = 1440
*M**_r_* = 341.82	*D*_x_ = 1.290 Mg m^−^^3^
Monoclinic, *C**c*	Mo *K*α radiation, λ = 0.71073 Å
Hall symbol: C -2yc	Cell parameters from 1325 reflections
*a* = 31.026 (6) Å	θ = 3.0–20.7°
*b* = 12.417 (2) Å	µ = 0.23 mm^−^^1^
*c* = 9.3209 (17) Å	*T* = 290 K
β = 101.423 (4)°	Block, colourless
*V* = 3519.8 (11) Å^3^	0.25 × 0.23 × 0.20 mm
*Z* = 8	

### Data collection

**Table:**

Bruker SMART CCD area-detector diffractometer	7013 independent reflections
Radiation source: fine-focus sealed tube	4933 reflections with *I* > 2σ(*I*)
graphite	*R*_int_ = 0.036
φ and ω scans	θ_max_ = 27.5°, θ_min_ = 1.8°
Absorption correction: multi-scan (*SADABS*; Sheldrick, 1996)	*h* = −38→40
*T*_min_ = 0.945, *T*_max_ = 0.956	*k* = −16→16
14797 measured reflections	*l* = −11→12

### Refinement

**Table:**

Refinement on *F*^2^	Secondary atom site location: difference Fourier map
Least-squares matrix: full	Hydrogen site location: inferred from neighbouring sites
*R*[*F*^2^ > 2σ(*F*^2^)] = 0.051	H-atom parameters constrained
*wR*(*F*^2^) = 0.094	*w* = 1/[σ^2^(*F*_o_^2^) + (0.0334*P*)^2^] where *P* = (*F*_o_^2^ + 2*F*_c_^2^)/3
*S* = 1.05	(Δ/σ)_max_ = 0.001
7013 reflections	Δρ_max_ = 0.31 e Å^−^^3^
435 parameters	Δρ_min_ = −0.13 e Å^−^^3^
2 restraints	Absolute structure: Flack (1983), 3002 Friedel pairs
Primary atom site location: structure-invariant direct methods	Flack parameter: 0.04 (5)

### Fractional atomic coordinates and isotropic or equivalent isotropic displacement parameters (Å^2^)

**Table:**

	*x*	*y*	*z*	*U*_iso_*/*U*_eq_	
Cl1	0.50000 (3)	0.64105 (6)	0.50836 (9)	0.0634 (2)	
Cl2	0.41803 (3)	0.49823 (7)	0.15694 (9)	0.0608 (2)	
O1	0.63439 (8)	0.1780 (2)	0.3091 (3)	0.0758 (7)	
O2	0.57673 (8)	0.53213 (18)	0.6705 (3)	0.0685 (7)	
O3	0.22780 (8)	0.6049 (2)	0.4959 (3)	0.0728 (7)	
O4	0.32828 (7)	0.55176 (18)	0.0346 (2)	0.0622 (6)	
N1	0.57108 (8)	0.3715 (2)	0.5510 (2)	0.0430 (6)	
N2	0.30973 (8)	0.64746 (17)	0.2214 (2)	0.0405 (6)	
C1	0.61735 (10)	0.2246 (3)	0.3972 (3)	0.0510 (8)	
C2	0.63947 (11)	0.3156 (3)	0.4873 (4)	0.0598 (9)	
H2A	0.6395	0.3782	0.4251	0.072*	
H2B	0.6698	0.2961	0.5258	0.072*	
C3	0.61746 (10)	0.3449 (3)	0.6139 (3)	0.0501 (8)	
H3	0.6313	0.4118	0.6560	0.060*	
C4	0.54533 (10)	0.2968 (2)	0.4414 (3)	0.0422 (7)	
H4	0.5391	0.3344	0.3471	0.051*	
C5	0.57193 (10)	0.1944 (2)	0.4222 (3)	0.0470 (8)	
H5	0.5761	0.1545	0.5146	0.056*	
C6	0.62226 (10)	0.2644 (3)	0.7379 (3)	0.0472 (8)	
C7	0.64743 (11)	0.1717 (3)	0.7463 (4)	0.0602 (9)	
H7	0.6631	0.1574	0.6730	0.072*	
C8	0.64984 (12)	0.1004 (3)	0.8605 (4)	0.0675 (10)	
H8	0.6670	0.0388	0.8640	0.081*	
C9	0.62698 (13)	0.1204 (3)	0.9686 (4)	0.0695 (10)	
H9	0.6279	0.0713	1.0446	0.083*	
C10	0.60264 (12)	0.2128 (3)	0.9652 (4)	0.0698 (11)	
H10	0.5875	0.2274	1.0397	0.084*	
C11	0.60076 (11)	0.2835 (3)	0.8510 (3)	0.0598 (9)	
H11	0.5844	0.3464	0.8501	0.072*	
C12	0.50134 (10)	0.2664 (2)	0.4800 (3)	0.0443 (7)	
C13	0.49875 (13)	0.2290 (3)	0.6171 (4)	0.0621 (10)	
H13	0.5242	0.2204	0.6880	0.074*	
C14	0.45792 (15)	0.2039 (3)	0.6495 (4)	0.0744 (12)	
H14	0.4562	0.1790	0.7423	0.089*	
C15	0.42043 (14)	0.2160 (3)	0.5452 (5)	0.0713 (11)	
H15	0.3932	0.1996	0.5672	0.086*	
C16	0.42293 (12)	0.2519 (3)	0.4098 (5)	0.0649 (9)	
H16	0.3974	0.2601	0.3392	0.078*	
C17	0.46276 (10)	0.2760 (2)	0.3765 (4)	0.0524 (8)	
H17	0.4640	0.2993	0.2826	0.063*	
C18	0.55669 (10)	0.4715 (2)	0.5775 (4)	0.0480 (8)	
C19	0.51356 (11)	0.5052 (2)	0.4795 (4)	0.0570 (9)	
H19A	0.4902	0.4589	0.4985	0.068*	
H19B	0.5158	0.4958	0.3780	0.068*	
C20	0.54732 (12)	0.1207 (3)	0.3038 (4)	0.0682 (10)	
H20A	0.5666	0.0647	0.2840	0.102*	
H20B	0.5371	0.1616	0.2163	0.102*	
H20C	0.5226	0.0893	0.3363	0.102*	
C21	0.32251 (10)	0.6907 (2)	0.3728 (3)	0.0411 (7)	
H21	0.3375	0.6327	0.4347	0.049*	
C22	0.28172 (11)	0.7229 (3)	0.4345 (3)	0.0492 (8)	
H22	0.2696	0.7884	0.3833	0.059*	
C23	0.24657 (10)	0.6372 (3)	0.4037 (4)	0.0510 (8)	
C24	0.23644 (11)	0.5946 (3)	0.2506 (3)	0.0520 (8)	
H24A	0.2054	0.6053	0.2109	0.062*	
H24B	0.2420	0.5177	0.2534	0.062*	
C25	0.26308 (10)	0.6470 (2)	0.1472 (3)	0.0446 (7)	
H25	0.2611	0.5977	0.0641	0.054*	
C26	0.24783 (10)	0.7553 (2)	0.0823 (3)	0.0470 (8)	
C27	0.21257 (12)	0.8110 (3)	0.1142 (4)	0.0613 (9)	
H27	0.1970	0.7822	0.1806	0.074*	
C28	0.20001 (14)	0.9089 (3)	0.0493 (4)	0.0825 (12)	
H28	0.1762	0.9455	0.0730	0.099*	
C29	0.22233 (16)	0.9526 (3)	−0.0500 (5)	0.0910 (14)	
H29	0.2141	1.0192	−0.0923	0.109*	
C30	0.25681 (15)	0.8972 (4)	−0.0860 (4)	0.0827 (12)	
H30	0.2719	0.9256	−0.1543	0.099*	
C31	0.26915 (12)	0.7997 (3)	−0.0212 (4)	0.0644 (10)	
H31	0.2924	0.7624	−0.0474	0.077*	
C32	0.35415 (10)	0.7850 (2)	0.3844 (3)	0.0427 (7)	
C33	0.39264 (11)	0.7851 (3)	0.4903 (3)	0.0525 (8)	
H33	0.3992	0.7256	0.5513	0.063*	
C34	0.42113 (12)	0.8708 (3)	0.5068 (4)	0.0656 (10)	
H34	0.4466	0.8688	0.5787	0.079*	
C35	0.41234 (14)	0.9586 (3)	0.4187 (5)	0.0741 (12)	
H35	0.4318	1.0164	0.4298	0.089*	
C36	0.37464 (15)	0.9612 (3)	0.3134 (5)	0.0749 (11)	
H36	0.3686	1.0211	0.2528	0.090*	
C37	0.34551 (13)	0.8754 (2)	0.2963 (4)	0.0597 (9)	
H37	0.3199	0.8785	0.2249	0.072*	
C38	0.33837 (10)	0.5885 (2)	0.1586 (3)	0.0439 (7)	
C39	0.38335 (10)	0.5695 (3)	0.2533 (3)	0.0506 (8)	
H39A	0.3802	0.5288	0.3395	0.061*	
H39B	0.3967	0.6382	0.2856	0.061*	
C40	0.29396 (13)	0.7496 (4)	0.5967 (4)	0.0788 (12)	
H40A	0.3109	0.6916	0.6477	0.118*	
H40B	0.2677	0.7594	0.6349	0.118*	
H40C	0.3110	0.8146	0.6098	0.118*	

### Atomic displacement parameters (Å^2^)

**Table:**

	*U*^11^	*U*^22^	*U*^33^	*U*^12^	*U*^13^	*U*^23^
Cl1	0.0701 (6)	0.0429 (4)	0.0813 (6)	0.0046 (4)	0.0246 (5)	0.0002 (4)
Cl2	0.0606 (5)	0.0579 (5)	0.0667 (5)	0.0203 (4)	0.0193 (4)	−0.0063 (4)
O1	0.0668 (16)	0.109 (2)	0.0558 (15)	0.0238 (15)	0.0222 (13)	−0.0105 (14)
O2	0.0717 (17)	0.0582 (14)	0.0703 (16)	−0.0018 (12)	0.0007 (14)	−0.0201 (13)
O3	0.0627 (17)	0.100 (2)	0.0621 (16)	−0.0086 (14)	0.0277 (14)	0.0125 (14)
O4	0.0593 (15)	0.0758 (15)	0.0515 (15)	0.0092 (12)	0.0109 (12)	−0.0225 (13)
N1	0.0408 (15)	0.0484 (15)	0.0405 (15)	0.0006 (12)	0.0094 (12)	−0.0013 (11)
N2	0.0452 (15)	0.0410 (13)	0.0375 (15)	0.0034 (12)	0.0134 (13)	−0.0056 (11)
C1	0.050 (2)	0.068 (2)	0.0367 (18)	0.0147 (17)	0.0124 (16)	0.0043 (16)
C2	0.049 (2)	0.075 (2)	0.060 (2)	0.0018 (17)	0.0217 (18)	0.0043 (18)
C3	0.0388 (19)	0.0572 (19)	0.055 (2)	−0.0025 (15)	0.0105 (16)	−0.0061 (16)
C4	0.0461 (18)	0.0433 (17)	0.0394 (18)	0.0002 (14)	0.0137 (15)	0.0031 (13)
C5	0.050 (2)	0.0525 (18)	0.0387 (18)	0.0098 (15)	0.0086 (15)	−0.0024 (14)
C6	0.0353 (17)	0.061 (2)	0.0430 (19)	0.0040 (15)	0.0025 (15)	−0.0085 (15)
C7	0.054 (2)	0.076 (2)	0.050 (2)	0.0153 (18)	0.0106 (17)	−0.0104 (18)
C8	0.069 (3)	0.069 (2)	0.060 (2)	0.019 (2)	0.002 (2)	−0.005 (2)
C9	0.065 (3)	0.083 (3)	0.055 (2)	0.006 (2)	−0.003 (2)	0.014 (2)
C10	0.056 (2)	0.108 (3)	0.046 (2)	0.019 (2)	0.0111 (18)	0.009 (2)
C11	0.056 (2)	0.076 (2)	0.047 (2)	0.0232 (18)	0.0069 (17)	−0.0036 (18)
C12	0.0487 (19)	0.0378 (15)	0.049 (2)	−0.0010 (14)	0.0154 (17)	−0.0046 (14)
C13	0.069 (3)	0.072 (2)	0.047 (2)	−0.0169 (19)	0.0184 (19)	0.0010 (18)
C14	0.099 (3)	0.080 (3)	0.053 (2)	−0.034 (2)	0.037 (3)	−0.011 (2)
C15	0.069 (3)	0.065 (2)	0.091 (3)	−0.024 (2)	0.044 (3)	−0.027 (2)
C16	0.051 (2)	0.0567 (19)	0.088 (3)	−0.0036 (17)	0.018 (2)	−0.013 (2)
C17	0.049 (2)	0.0485 (18)	0.061 (2)	0.0009 (15)	0.0143 (19)	0.0016 (16)
C18	0.051 (2)	0.0435 (18)	0.053 (2)	−0.0006 (15)	0.0169 (17)	0.0006 (15)
C19	0.061 (2)	0.0439 (17)	0.068 (2)	0.0047 (15)	0.0152 (19)	−0.0038 (16)
C20	0.075 (3)	0.063 (2)	0.066 (2)	0.0087 (19)	0.013 (2)	−0.0142 (19)
C21	0.0509 (19)	0.0379 (15)	0.0357 (17)	0.0038 (14)	0.0117 (14)	0.0004 (13)
C22	0.055 (2)	0.0528 (19)	0.0436 (19)	0.0015 (16)	0.0183 (16)	−0.0069 (15)
C23	0.0430 (19)	0.058 (2)	0.055 (2)	0.0062 (16)	0.0172 (17)	0.0040 (17)
C24	0.049 (2)	0.0507 (18)	0.058 (2)	−0.0066 (15)	0.0133 (17)	−0.0029 (16)
C25	0.0441 (19)	0.0495 (18)	0.0411 (18)	−0.0006 (14)	0.0105 (15)	−0.0066 (15)
C26	0.049 (2)	0.0549 (18)	0.0374 (18)	0.0057 (15)	0.0101 (15)	−0.0041 (14)
C27	0.056 (2)	0.074 (2)	0.057 (2)	0.0151 (18)	0.0185 (18)	0.0047 (19)
C28	0.090 (3)	0.081 (3)	0.080 (3)	0.041 (2)	0.025 (2)	0.004 (2)
C29	0.125 (4)	0.069 (3)	0.080 (3)	0.035 (3)	0.023 (3)	0.020 (2)
C30	0.103 (3)	0.088 (3)	0.064 (3)	0.015 (3)	0.033 (2)	0.022 (2)
C31	0.070 (2)	0.072 (2)	0.056 (2)	0.0188 (19)	0.0251 (19)	0.0126 (19)
C32	0.054 (2)	0.0362 (16)	0.0416 (18)	0.0032 (14)	0.0196 (16)	−0.0060 (13)
C33	0.058 (2)	0.0447 (19)	0.055 (2)	0.0006 (16)	0.0115 (18)	−0.0064 (15)
C34	0.059 (2)	0.057 (2)	0.079 (3)	−0.0010 (18)	0.009 (2)	−0.021 (2)
C35	0.071 (3)	0.050 (2)	0.108 (3)	−0.015 (2)	0.034 (3)	−0.027 (2)
C36	0.093 (3)	0.042 (2)	0.097 (3)	−0.002 (2)	0.034 (3)	0.009 (2)
C37	0.069 (2)	0.0425 (18)	0.069 (2)	0.0015 (17)	0.0165 (19)	0.0029 (17)
C38	0.047 (2)	0.0411 (16)	0.0456 (19)	0.0016 (14)	0.0133 (16)	−0.0030 (15)
C39	0.055 (2)	0.0508 (19)	0.0492 (19)	0.0109 (15)	0.0166 (17)	−0.0045 (15)
C40	0.077 (3)	0.106 (3)	0.059 (2)	−0.009 (2)	0.029 (2)	−0.026 (2)

### Geometric parameters (Å, °)

**Table:**

Cl1—C19	1.771 (3)	C18—C19	1.522 (4)
Cl2—C39	1.769 (3)	C19—H19A	0.97
O1—C1	1.208 (3)	C19—H19B	0.97
O2—C18	1.221 (4)	C20—H20A	0.96
O3—C23	1.198 (3)	C20—H20B	0.96
O4—C38	1.224 (3)	C20—H20C	0.96
N1—C18	1.360 (4)	C21—C32	1.518 (4)
N1—C3	1.480 (4)	C21—C22	1.543 (4)
N1—C4	1.489 (4)	C21—H21	0.98
N2—C38	1.368 (3)	C22—C23	1.510 (4)
N2—C25	1.475 (4)	C22—C40	1.521 (4)
N2—C21	1.489 (3)	C22—H22	0.98
C1—C2	1.491 (5)	C23—C24	1.496 (4)
C1—C5	1.520 (4)	C24—C25	1.534 (4)
C2—C3	1.520 (4)	C24—H24A	0.97
C2—H2A	0.97	C24—H24B	0.97
C2—H2B	0.97	C25—C26	1.511 (4)
C3—C6	1.513 (4)	C25—H25	0.98
C3—H3	0.98	C26—C27	1.376 (4)
C4—C12	1.526 (4)	C26—C31	1.387 (4)
C4—C5	1.546 (4)	C27—C28	1.379 (5)
C4—H4	0.98	C27—H27	0.93
C5—C20	1.517 (4)	C28—C29	1.373 (6)
C5—H5	0.98	C28—H28	0.93
C6—C11	1.375 (4)	C29—C30	1.368 (5)
C6—C7	1.384 (4)	C29—H29	0.93
C7—C8	1.375 (5)	C30—C31	1.373 (5)
C7—H7	0.93	C30—H30	0.93
C8—C9	1.365 (5)	C31—H31	0.93
C8—H8	0.93	C32—C37	1.385 (4)
C9—C10	1.371 (5)	C32—C33	1.390 (4)
C9—H9	0.93	C33—C34	1.373 (4)
C10—C11	1.372 (5)	C33—H33	0.93
C10—H10	0.93	C34—C35	1.360 (5)
C11—H11	0.93	C34—H34	0.93
C12—C13	1.377 (4)	C35—C36	1.370 (5)
C12—C17	1.386 (4)	C35—H35	0.93
C13—C14	1.394 (5)	C36—C37	1.386 (5)
C13—H13	0.93	C36—H36	0.93
C14—C15	1.369 (5)	C37—H37	0.93
C14—H14	0.93	C38—C39	1.515 (4)
C15—C16	1.354 (5)	C39—H39A	0.97
C15—H15	0.93	C39—H39B	0.97
C16—C17	1.366 (5)	C40—H40A	0.96
C16—H16	0.93	C40—H40B	0.96
C17—H17	0.93	C40—H40C	0.96
			
C18—N1—C3	117.4 (3)	C5—C20—H20C	109.5
C18—N1—C4	122.9 (2)	H20A—C20—H20C	109.5
C3—N1—C4	118.7 (2)	H20B—C20—H20C	109.5
C38—N2—C25	117.2 (2)	N2—C21—C32	113.1 (2)
C38—N2—C21	121.6 (2)	N2—C21—C22	111.3 (2)
C25—N2—C21	119.9 (2)	C32—C21—C22	109.9 (2)
O1—C1—C2	122.1 (3)	N2—C21—H21	107.4
O1—C1—C5	122.0 (3)	C32—C21—H21	107.4
C2—C1—C5	116.0 (3)	C22—C21—H21	107.4
C1—C2—C3	113.0 (3)	C23—C22—C40	111.8 (3)
C1—C2—H2A	109.0	C23—C22—C21	111.2 (2)
C3—C2—H2A	109.0	C40—C22—C21	111.4 (3)
C1—C2—H2B	109.0	C23—C22—H22	107.4
C3—C2—H2B	109.0	C40—C22—H22	107.4
H2A—C2—H2B	107.8	C21—C22—H22	107.4
N1—C3—C6	113.0 (3)	O3—C23—C24	121.7 (3)
N1—C3—C2	107.3 (3)	O3—C23—C22	122.3 (3)
C6—C3—C2	116.3 (3)	C24—C23—C22	116.0 (3)
N1—C3—H3	106.6	C23—C24—C25	114.3 (3)
C6—C3—H3	106.6	C23—C24—H24A	108.7
C2—C3—H3	106.6	C25—C24—H24A	108.7
N1—C4—C12	112.0 (2)	C23—C24—H24B	108.7
N1—C4—C5	111.4 (2)	C25—C24—H24B	108.7
C12—C4—C5	110.3 (2)	H24A—C24—H24B	107.6
N1—C4—H4	107.6	N2—C25—C26	112.6 (2)
C12—C4—H4	107.6	N2—C25—C24	107.9 (2)
C5—C4—H4	107.6	C26—C25—C24	117.7 (3)
C20—C5—C1	112.7 (3)	N2—C25—H25	105.9
C20—C5—C4	112.1 (3)	C26—C25—H25	105.9
C1—C5—C4	110.3 (2)	C24—C25—H25	105.9
C20—C5—H5	107.1	C27—C26—C31	117.4 (3)
C1—C5—H5	107.1	C27—C26—C25	124.0 (3)
C4—C5—H5	107.1	C31—C26—C25	118.5 (3)
C11—C6—C7	117.0 (3)	C26—C27—C28	121.1 (3)
C11—C6—C3	118.8 (3)	C26—C27—H27	119.5
C7—C6—C3	124.2 (3)	C28—C27—H27	119.5
C8—C7—C6	121.5 (3)	C29—C28—C27	120.5 (4)
C8—C7—H7	119.2	C29—C28—H28	119.7
C6—C7—H7	119.2	C27—C28—H28	119.7
C9—C8—C7	119.9 (3)	C30—C29—C28	119.3 (4)
C9—C8—H8	120.0	C30—C29—H29	120.3
C7—C8—H8	120.0	C28—C29—H29	120.3
C8—C9—C10	119.9 (4)	C29—C30—C31	119.9 (4)
C8—C9—H9	120.0	C29—C30—H30	120.0
C10—C9—H9	120.0	C31—C30—H30	120.0
C9—C10—C11	119.5 (3)	C30—C31—C26	121.8 (3)
C9—C10—H10	120.2	C30—C31—H31	119.1
C11—C10—H10	120.2	C26—C31—H31	119.1
C10—C11—C6	122.1 (3)	C37—C32—C33	117.3 (3)
C10—C11—H11	118.9	C37—C32—C21	122.4 (3)
C6—C11—H11	118.9	C33—C32—C21	120.2 (3)
C13—C12—C17	118.3 (3)	C34—C33—C32	121.6 (3)
C13—C12—C4	121.4 (3)	C34—C33—H33	119.2
C17—C12—C4	120.3 (3)	C32—C33—H33	119.2
C12—C13—C14	119.9 (4)	C35—C34—C33	120.4 (4)
C12—C13—H13	120.0	C35—C34—H34	119.8
C14—C13—H13	120.0	C33—C34—H34	119.8
C15—C14—C13	120.1 (3)	C34—C35—C36	119.5 (3)
C15—C14—H14	119.9	C34—C35—H35	120.3
C13—C14—H14	119.9	C36—C35—H35	120.3
C16—C15—C14	120.0 (4)	C35—C36—C37	120.6 (4)
C16—C15—H15	120.0	C35—C36—H36	119.7
C14—C15—H15	120.0	C37—C36—H36	119.7
C15—C16—C17	120.4 (4)	C32—C37—C36	120.6 (4)
C15—C16—H16	119.8	C32—C37—H37	119.7
C17—C16—H16	119.8	C36—C37—H37	119.7
C16—C17—C12	121.2 (3)	O4—C38—N2	122.5 (3)
C16—C17—H17	119.4	O4—C38—C39	121.7 (3)
C12—C17—H17	119.4	N2—C38—C39	115.8 (3)
O2—C18—N1	123.6 (3)	C38—C39—Cl2	111.4 (2)
O2—C18—C19	121.4 (3)	C38—C39—H39A	109.3
N1—C18—C19	115.1 (3)	Cl2—C39—H39A	109.3
C18—C19—Cl1	112.2 (2)	C38—C39—H39B	109.3
C18—C19—H19A	109.2	Cl2—C39—H39B	109.3
Cl1—C19—H19A	109.2	H39A—C39—H39B	108.0
C18—C19—H19B	109.2	C22—C40—H40A	109.5
Cl1—C19—H19B	109.2	C22—C40—H40B	109.5
H19A—C19—H19B	107.9	H40A—C40—H40B	109.5
C5—C20—H20A	109.5	C22—C40—H40C	109.5
C5—C20—H20B	109.5	H40A—C40—H40C	109.5
H20A—C20—H20B	109.5	H40B—C40—H40C	109.5
			
O1—C1—C2—C3	−166.7 (3)	C38—N2—C21—C32	73.0 (3)
C5—C1—C2—C3	13.7 (4)	C25—N2—C21—C32	−120.2 (3)
C18—N1—C3—C6	109.4 (3)	C38—N2—C21—C22	−162.7 (2)
C4—N1—C3—C6	−81.7 (3)	C25—N2—C21—C22	4.1 (3)
C18—N1—C3—C2	−121.1 (3)	N2—C21—C22—C23	45.2 (3)
C4—N1—C3—C2	47.8 (3)	C32—C21—C22—C23	171.3 (3)
C1—C2—C3—N1	−57.4 (3)	N2—C21—C22—C40	170.7 (3)
C1—C2—C3—C6	70.2 (4)	C32—C21—C22—C40	−63.2 (3)
C18—N1—C4—C12	−62.3 (3)	C40—C22—C23—O3	7.8 (5)
C3—N1—C4—C12	129.4 (3)	C21—C22—C23—O3	133.1 (3)
C18—N1—C4—C5	173.6 (3)	C40—C22—C23—C24	−172.2 (3)
C3—N1—C4—C5	5.3 (3)	C21—C22—C23—C24	−46.9 (4)
O1—C1—C5—C20	−13.2 (4)	O3—C23—C24—C25	178.8 (3)
C2—C1—C5—C20	166.4 (3)	C22—C23—C24—C25	−1.2 (4)
O1—C1—C5—C4	−139.4 (3)	C38—N2—C25—C26	−111.9 (3)
C2—C1—C5—C4	40.2 (4)	C21—N2—C25—C26	80.7 (3)
N1—C4—C5—C20	−176.3 (3)	C38—N2—C25—C24	116.5 (3)
C12—C4—C5—C20	58.6 (3)	C21—N2—C25—C24	−50.8 (3)
N1—C4—C5—C1	−49.8 (3)	C23—C24—C25—N2	48.3 (3)
C12—C4—C5—C1	−174.9 (2)	C23—C24—C25—C26	−80.4 (4)
N1—C3—C6—C11	−51.3 (4)	N2—C25—C26—C27	−124.2 (3)
C2—C3—C6—C11	−176.0 (3)	C24—C25—C26—C27	2.3 (4)
N1—C3—C6—C7	129.2 (3)	N2—C25—C26—C31	59.0 (4)
C2—C3—C6—C7	4.5 (4)	C24—C25—C26—C31	−174.5 (3)
C11—C6—C7—C8	1.8 (5)	C31—C26—C27—C28	−2.2 (5)
C3—C6—C7—C8	−178.7 (3)	C25—C26—C27—C28	−179.0 (3)
C6—C7—C8—C9	0.2 (5)	C26—C27—C28—C29	0.5 (6)
C7—C8—C9—C10	−1.8 (5)	C27—C28—C29—C30	1.1 (7)
C8—C9—C10—C11	1.4 (6)	C28—C29—C30—C31	−1.0 (7)
C9—C10—C11—C6	0.7 (6)	C29—C30—C31—C26	−0.7 (6)
C7—C6—C11—C10	−2.2 (5)	C27—C26—C31—C30	2.3 (5)
C3—C6—C11—C10	178.3 (3)	C25—C26—C31—C30	179.3 (3)
N1—C4—C12—C13	−50.8 (4)	N2—C21—C32—C37	53.2 (4)
C5—C4—C12—C13	74.0 (3)	C22—C21—C32—C37	−71.8 (4)
N1—C4—C12—C17	129.3 (3)	N2—C21—C32—C33	−129.5 (3)
C5—C4—C12—C17	−105.9 (3)	C22—C21—C32—C33	105.5 (3)
C17—C12—C13—C14	−1.3 (5)	C37—C32—C33—C34	−0.3 (4)
C4—C12—C13—C14	178.8 (3)	C21—C32—C33—C34	−177.7 (3)
C12—C13—C14—C15	0.3 (5)	C32—C33—C34—C35	−0.2 (5)
C13—C14—C15—C16	0.3 (5)	C33—C34—C35—C36	0.3 (5)
C14—C15—C16—C17	0.1 (5)	C34—C35—C36—C37	0.1 (6)
C15—C16—C17—C12	−1.1 (5)	C33—C32—C37—C36	0.7 (5)
C13—C12—C17—C16	1.8 (4)	C21—C32—C37—C36	178.0 (3)
C4—C12—C17—C16	−178.3 (3)	C35—C36—C37—C32	−0.6 (6)
C3—N1—C18—O2	−14.2 (4)	C25—N2—C38—O4	12.3 (4)
C4—N1—C18—O2	177.4 (3)	C21—N2—C38—O4	179.5 (3)
C3—N1—C18—C19	164.4 (3)	C25—N2—C38—C39	−166.5 (2)
C4—N1—C18—C19	−4.0 (4)	C21—N2—C38—C39	0.6 (4)
O2—C18—C19—Cl1	4.6 (4)	O4—C38—C39—Cl2	4.0 (4)
N1—C18—C19—Cl1	−174.0 (2)	N2—C38—C39—Cl2	−177.1 (2)
